# Autopsy Tool in Unknown Diseases: The Experience with Coronaviruses (SARS-CoV, MERS-CoV, SARS-CoV-2)

**DOI:** 10.3390/medicina57040309

**Published:** 2021-03-25

**Authors:** Francesco Sessa, Monica Salerno, Cristoforo Pomara

**Affiliations:** 1 Department of Clinical and Experimental Medicine, Institute of Legal Medicine, University of Foggia, 71122 Foggia, Italy; 2 Department of Medical, Surgical and Advanced Technologies “G.F. Ingrassia”, Institute of Legal Medicine, University of Catania, 95121 Catania, Italy; monica.salerno@unict.it

**Keywords:** autopsy, unknown diseases, SARS-CoV, MERS-CoV, SARS-CoV-2, COVID-19

## Abstract

In the last two decades, three unknown pathogens have caused outbreaks, generating severe global health concerns. In 2003, after nucleic acid genotyping, a new virus was named severe acute respiratory syndrome coronavirus (SARS-CoV). After nine years, another coronavirus emerged in the middle east and was named MERS-CoV (Middle East Respiratory Syndrome—Coronavirus). Finally, in December 2019, a new unknown coronavirus was isolated from a cluster of patients and was named SARS-CoV-2 (COVID-19, coronavirus disease 2019). This review aims to propose a complete overview of autopsy in the three coronaviruses over the past two decades, showing its pivotal role in the management of unknown diseases. A total of 116 studies fulfilled the inclusion criteria: 14 studies were collected concerning SARS-CoV (87 autopsy reports, from Asian and American countries), 2 studies for MERS-CoV (2 autopsy reports, from Middle-East Asian countries), and 100 studies on SARS-CoV-2 (930 autopsy reports). Analyzing the data obtained on COVID-19, based on the country criterion, a large number of post-mortem investigation were performed in European countries (580 reports), followed by American countries (251 reports). It is interesting to note that no data were found from the Oceanic countries, maybe because of the minor involvement of the outbreak. In all cases, autopsy provided much information about each unknown coronavirus. Despite advanced technologies in the diagnostic fields, to date, autopsy remains the gold standard method to understand the biological features and the pathogenesis of unknown infections, especially when awareness of a pathogen is restricted and the impact on the healthcare system is substantial. The knowledge gained through this technique may positively influence therapeutic strategies, ultimately reducing mortality.

## 1. Introduction

In the last two decades, three unknown pathogens have caused outbreaks, generating severe global health concerns. All agents are coronaviruses, composed of an envelope, a single strand of RNA (positive-sense) associated with a nucleoprotein within a capsid [1].

In 2003, after nucleic acid genotyping, a new virus was named severe acute respiratory syndrome coronavirus (SARS-CoV). In symptomatic patients, this infection generates fever, SARS, and, in severe cases, pneumonia and lower respiratory symptoms such as cough and dyspnea [2]. The SARS-CoV epidemic emerged in China, spreading to many countries in South-East Asia, North America, Europe, and South Africa. Transmission occurred mainly from person to person through droplets during coughing or sneezing, through personal contact, or by touching contaminated surfaces. Healthcare workers were the major group of persons exposed to SARS-CoV infection risk, particularly when suggested precautions and safety procedures were not respected. Based on the international data, SARS-CoV infected more than 8000 people causing 774 deaths with an estimated mortality rate of 9.5% [3].

After nine years, another coronavirus emerged in the Middle East and was named MERS-CoV (Middle East Respiratory Syndrome–Coronavirus). The symptoms were not specific, even if various patients manifested severe acute respiratory distress. Each patient affected by this coronavirus was linked to subjects in or near the Arabian Peninsula. As occurred in the case of the SARS-CoV infection, healthcare personnel were the group most exposed to this pathogen. Even if MERS-CoV has a low transmission rate, it has a mortality rate much higher than SARS-CoV, about 35% [4]. From 2012 to 15 January 2020, the total number of MERS-CoV cases confirmed in the laboratory and reported worldwide to the World Health Organization (WHO) was 2506, with 862 associated deaths [5].

Finally, in December 2019, a new unknown coronavirus was reported in China. It was isolated from a cluster of patients and it was named SARS-CoV-2 (COVID-19) from the WHO in February 2020. The symptoms are fever, dry cough, and shortness of breath and, in the most severe cases, SARS [6]. Even if the fatality rate is about 2–3% (lower than MERS), it has caused many more deaths than its predecessors, considering that for its diffusion it has been declared a pandemic. Moreover, severe containment measures have been adopted worldwide in order to avoid the collapse of healthcare systems (limiting human circulation, closing airports, train stations, and applying the so-called “lockdown”). The scientific community has worked hard to clarify the major aspects of this new infection, considering that, in the last decades, COVID-19 is a major concern for healthcare systems worldwide. Different scientific organizations provided guidelines and recommendations including safety measures to perform post-mortem investigations safely and effectively [7,8,9]. Nevertheless, in the first phase of this pandemic, the value of autopsy was underestimated, and only a few autopsies were performed. Undoubtedly, this choice (named “lockdown of science” [10]) is an important error in the management of the COVID-19 infection, delaying the knowledge about this new pathogen and consequently, correct therapeutic approaches [11].

Based on the previous unknown infections, such as SARS-CoV and MERS-CoV, it is important to remark that the post-mortem investigation may be considered the gold standard method to understand pathophysiological mechanisms, contributing to clarify morphological and virology features, suggesting unexplored therapeutic approaches and new frontiers of research [12,13,14]. For example, translating the scientific knowledge acquired from its predecessors (SARS-CoV and MERS-CoV), it has been established that the ACE2 (angiotensin converting enzyme ace 2) receptors represent the entrance doors in the host cells for SARS-CoV-2 [15].

In this regard, this review aims to propose a complete overview of autopsy in the three coronaviruses (MERS-CoV, SARS-CoV, and SARS-CoV-2) over the past two decades, showing its pivotal role in the management of unknown diseases.

## 2. Materials and Methods

### 2.1. Eligibility Criteria

We included all case reports, case series, retrospective and prospective studies, letters to the editors, and reviews that focused on MERS-CoV, SARS-CoV, SARS-CoV-2, and autopsy. The search was limited to human studies.

### 2.2. Search Criteria and Critical Appraisal

**Figure 1:**
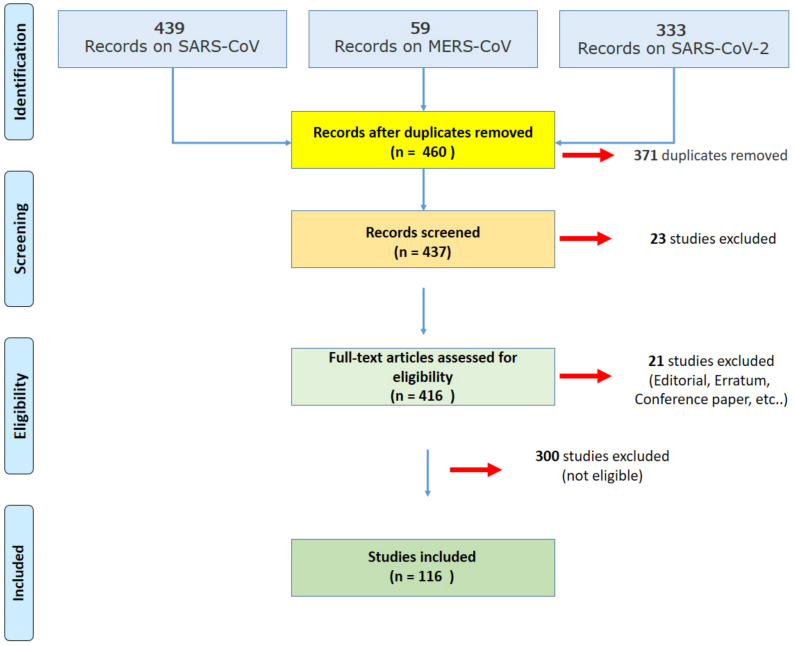
The PRISMA (Preferred Reporting Items for Systematic Reviews and Meta-Analyses) strategy used for the literature review.

A systematic literature search and a critical appraisal of the collected studies were conducted. An electronic search of ScienceDirect Scopus from the inception of this database to the 31 January 2021 was performed. Search terms were “AUTOPSY” OR “POST-MORTEM” AND “MERS” OR “SARS-CoV” OR “SARS-CoV-2” OR “COVID-19” in the title, abstract, and keywords. Cases in which autopsy was not performed were excluded because they did not meet the inclusion criteria. The bibliographies of all selected papers were examined and cross-referenced for further relevant literature. A methodological appraisal of each study was conducted according to the PRISMA (Preferred Reporting Items for Systematic Reviews and Meta-Analyses) standards (Figure 1). This study was exempt from institutional review board approval as it did not involve human subjects.

## 3. Results

### 3.1. Search Results and Included Studies

An appraisal based on titles and abstracts as well as a visual search of reference lists were carried out. The reference lists of all selected articles were reviewed to detect still unidentified literature. A total of 116 studies fulfilled the inclusion criteria: 14 studies were collected concerning SARS-CoV, 2 studies on MERS-CoV, and 100 studies on SARS-CoV-2.

Considering the selected articles on SARS-CoV, the pooled dataset obtained from 87 autopsies was analyzed. In the case of MERS-CoV, only 2 case reports were collected. Finally, as concerns SARS-COV-2, a pooled dataset of 930 autopsies was analyzed.

### 3.2. Autopsy and SARS-CoV

Table 1 shows the data extracted from the included studies about the SARS-CoV infection, summarized in study source, country, autopsy number, and diagnostic technique.

The SARS-CoV disease spread to 29 countries on 5 continents, even if the Asian countries and Canada represented the areas with the highest number of cases [30]. In consideration of this, as summarized in Table 1, all collected articles analyzed the post-mortem dataset collected in the same countries.

He et al. [19] analyzed the presence and distribution of SARS-CoV in autopsy tissues through the immunohistochemical technique, confirming the presence of the virus in all analyzed tissues (lungs, spleen, lymph nodes, brain, pituitary gland, heart, liver, kidney, pancreas, trachea, esophagus, gastrointestinal tract, adrenal glands, parathyroid glands, skin, and bone marrow). Similar results were obtained in other papers [16] and with other techniques, such as the in situ hybridization technique [20].

As described by Franks et al. [26], the predominant pattern of lung injury in the cases of subjects who died from SARS-CoV infection was diffuse alveolar damage (DAD). They reported that the histology findings varied based on the duration of illness: in cases with 10 or fewer days’ duration, acute-phase DAD, airspace edema, and bronchiolar fibrin were described. On the other hand, the cases of more than 10 days’ duration exhibited organizing-phase DAD, type II pneumocyte hyperplasia, squamous metaplasia, multinucleated giant cells, and acute bronchopneumonia. Moreover, lung fibrosis with alveolar septa and interstitium thickening were detected [22]. Similar results were reported by other papers [25,28,29].

Nicholls et al. [21] provided evidence on the damage that occurred in the other tissues. Particularly, they reported atrophy of the white pulp of the spleen. Lymphoid depletion was reported by Hsiao et al. [29], and the same authors did not detect pathological findings regarding the gastrointestinal tract and kidneys. The results from the gastrointestinal tract were not in agreement with the results collected by Shi et al. [23], who described the presence of the viral infection at this level, with the generation of classical adverse effects, and they found SARS-CoV in the stools of the patients affected by the same infection.

He et al. [24] published a paper in 2006 studying the post-mortem samples and clarified several important questions about the SARS-CoV infection, describing that ACE2-expressing cells were the primary targets for the SARS-CoV infection and, after the infection, the same cells produced high levels of pro-inflammatory cytokines, generating immuno-mediated damage. Similarly, in 2009, Oudit et al. [17] described that the myocardial dysfunction found in SARS-CoV subjects benefited from the presence of ACE2 receptors.

### 3.3. Autopsies and MERS

MERS-CoV was identified for the first time in a male subject with severe pneumonia in 2012. Subsequently, this infection has been detected in more than 1500 individuals, showing different degrees of severity, ranging from asymptomatic to severe status, leading to fatal pneumonia in several cases. The scientific community avoided performing timely autopsy studies on MERS victims. This mistake limited the knowledge about the pathogenesis of MERS-CoV [31]. In this scenario, with very few data about the post-mortem investigation, several animal models have been used to clarify the major aspects of this unknown disease [32].

The following data were extracted from the included studies about the MERS-CoV infection, and Table 2 summarizes the main data.

Based on this literature review, only two papers have been published regarding autopsy and MERS-CoV-2. It is interesting to note that the first study was published in 2016 [33], four years after the spread of this infection, and the last study was published in 2018, even if it refers to an autopsy performed in 2014 [34]. Based on WHO data, this infection is still ongoing in Middle East countries, even if the cases are sporadic.

As reported by Ng et al. [33], the histopathological data of MERS-CoV in humans are insufficiently described, for this reason, they explored the histological and ultrastructural changes involving various organs of a MERS-CoV patient. In their case, they focused on the heart, brain, lung, kidney, liver, and skeletal muscles, showing necrotizing pneumonia, DAD, acute kidney injury, portal and lobular hepatitis, and myositis with atrophic muscle changes. However, no histological modifications were detected on the brain and heart. Based on this study, the pulmonary and extrapulmonary involvement of MERS-CoV infection was proven, providing, for the first time, evidence about the presence of the virus in human renal tissue.

In agreement with Ng et al., Alsaad et al. [34] reported that the main finding of their study was DAD; moreover, they detected the presence of cardiac fibrosis. In contrast with the previous study, they did not detect the presence of the viral antigen at the level of the kidney.

In conclusion, few studies have been published on the discussed theme. Undoubtedly, the lack of autopsy findings has reduced the knowledge about MERS-CoV.

### 3.4. Autopsies and SARS-CoV-2

Considering the pandemic diffusion of the SARS-CoV-2 infection, numerous autopsy studies have been performed. It is important to remark that in the first phase of infection, several countries discouraged autopsies, limiting the post-mortem investigation only to forensic cases [10]. However, in the second phase, after alarming reports from scientists [11], many autopsies were performed, clarifying several pathological aspects of COVID-19. Viral detection in different tissues has frequently been described: the most common method used during post-mortem investigations is immunohistochemistry [35], using an antibody versus viral spike protein.

The following data were extracted from the included studies about the SARS-CoV-2 infection. Table 3 summarizes the main data.

Based on the country criterion, 95 autopsy investigations were performed in Asian countries, 580 in European countries, 251 in American countries, and 4 cases in African countries. It is interesting to note that no autopsy investigations were described from the Oceanic countries, maybe because of the minor involvement of the outbreak. The countries with the greatest number of autopsy reports are the USA (227), followed by Italy (212 autopsy reports) and Germany (188 reports). Italy was the first European country involved in the infection spread, even if autopsies were strongly discouraged by the Health Minister in the first phase of infection [136]. However, in several areas of Germany, autopsy was considered mandatory in cases of confirmed or suspected COVID-19 death. For example, one of the most important findings defined thanks to autopsy procedures was the identification of a pro-coagulative status in COVID-19 patients, identifying macroscopic and/or microscopic thrombi at the pulmonary level [79,111,132]. These findings undoubtfully improved the therapy of COVID-19 inpatients.

**Figure 2:**
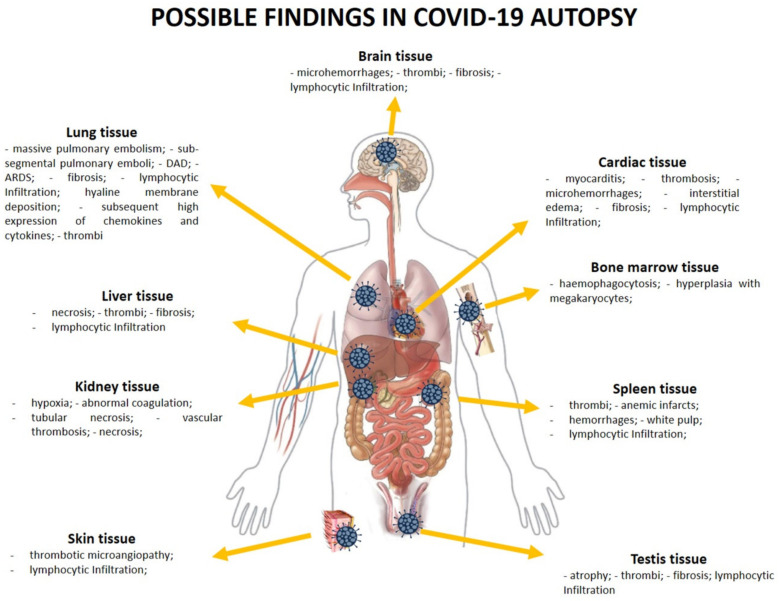
The possible findings in a COVID-19 autopsy (abbreviations: acute respiratory distress syndrome, ARDS; diffuse alveolar damages, DAD).

In Figure 2 and in the following subsections, the findings that may be found in different tissues at the autopsy examination in COVID-19 patients are summarized.

#### 3.4.1. SARS-CoV-2 and Lung Tissue

As previously described, COVID-19 predominantly involves the lungs, generating, in several patients, acute respiratory insufficiency. Different types of post-mortem investigations have been performed, ranging from biopsies to full autopsies [47]. In particular, pulmonary pathology was heterogeneous, reflecting radiological findings of patchy ground-glass opacities, and the histology of the lung parenchyma showed a variety of findings from exudative hyaline membrane disease to organized pneumonia, whereas some areas were histologically normal.

At the gross examination, the lungs were frequently described as heavy, congested, and edematous, frequently with a focal bilateral consolidation [57,72,100,116]. The pleura could be found inconspicuous [86]. This finding is more debated than reported, even if the pleural involvement in COVID-19 patients has been demonstrated in radiological and histological studies [137,138]. In several cases, massive pulmonary embolism was described as the main cause of death [130]. Sub-segmental pulmonary emboli was also frequently found to cause lobar infarction [48,116,121]. Histologically, the lungs presented the typical changes of the acute exudative phase of bilateral DAD [48,84,116] with the clinical presentation of acute respiratory distress syndrome (ARDS) [106,114]. COVID-19 patients could develop the fibrosing pattern as a consequence of DAD [37]. In other cases, interstitial pulmonary lymphoid infiltrates and enlarged atypical pneumocytes have been reported, without DAD [88]. Analyzing the vascular modification, in COVID-19 vascular injury is a distinctive pathological feature: thrombotic microangiopathy coexists with other lesions such as endothelialitis and pulmonary angiogenesis. Distinctive findings of the COVID-19 damage at the level of the lung included the presence of type II pneumocytes with nucleomegaly and prominent nucleoli, combined with an accumulation of macrophages, lymphocytes, and multinucleated giant cells [67,72,78,106,116,121]. Different infiltrated lymphocytes were also found [57], with the presence of abundant megakaryocytes [99]. Moreover, neutrophil extracellular traps (NETs) can contribute to inflammation-associated lung damage, thrombosis, and fibrosis [52]. Thrombi in pre- and post-capillary vessels were frequently described [47,50,62,78,113,120,135]: thrombi usually appeared hetero-synchronous, at different stages of organization [48,66,79,83]. It is interesting to note that a higher presence of ACE2 in alveolar epithelial cells and capillary endothelial cells facilitate the entrance of SARS-CoV-2 [62], generating a subsequent high expression of chemokines and cytokines (IL(interleukin) -6, IL10, TNF (Tumor Necrosis Factor)-α) [57]. The pro-thrombotic state is usually promoted by a high expression of IL-6, followed by ICAM-1 (Intercellular Adhesion Molecule 1) expression and endotheliites, generating a systemic inflammatory response syndrome [108]. Hellman et al. described the presence of glycosaminoglycan hyaluronan (HA) in the alveolar spaces in lung tissue, suggesting new treatment options in severe COVID-19 [97].

#### 3.4.2. SARS-CoV-2 and Cardiac Tissue

The presence of the SARS-CoV-2 in cardiac tissue is frequently reported in the post-mortem investigations. An important point in the evaluation of heart tissue concerns the consideration of tissue damage: considering that the subjects who died with/from COVID-19 have more than one comorbidity [10], it is very difficult to establish if the observed lesion is related to the infection or to pre-existing conditions. This thematic is very important, considering that the comorbidities, such as hypertension, can frequently generate tissue damage. At the gross examination, the heart showed increased size and weight, hypertrophy, and dilation of the left and right atria and ventricles. The myocardium appeared pale and flaccid, and the endocardium showed punctuated petechial hemorrhages [81]. Considering the high prevalence of subjects with comorbidities such as hypertension, in the post-mortem examination, left ventricular hypertrophy, dilated cardiomyopathy, and hypertrophic cardiomyopathy were commonly described [100,121]. A variable pattern of cardiomyocyte injury was consistently observed, ranging from the absence of cell death and subcellular changes to intracellular edema and sarcomere disruption [76]. Moreover, in different reports, lymphocytic viral myocarditis was reported, and these findings were morphologically and immunohistochemically confirmed in the post-mortem investigation. In several cases, myocarditis was described in COVID-19 patients—it developed in the presence of coronary artery disease and may be concurrent with lymphocytic endo-pericarditis [61,92,105]. At the level of the myocardium, thrombosis, microhemorrhages, and interstitial edema have frequently been shown [50,89]. It is important to highlight that fulminant myocarditis may be found in cases of quick exitus for COVID-19 patients [95,112,113]. Moreover, in a multicenter study focusing on cardiac tissue, Basso et al. [87] reported macrophage infiltration in many cases; nevertheless, they concluded that this condition may be related to underlying diseases rather than COVID-19.

In myocardial tissue, signs of viral presence were also observed [73], and these findings suggest the need for cardiological surveillance in COVID-19 survivors.

#### 3.4.3. SARS-CoV-2 in Spleen and Bone Marrow Tissues

**Figure 3:**
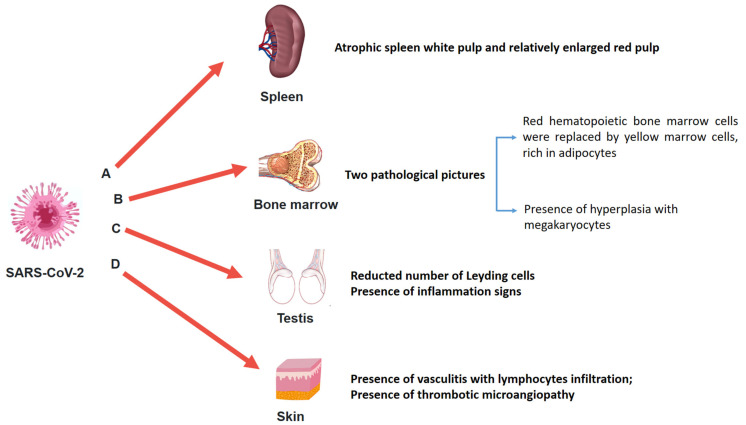
Different organs such as spleen, bone marrow, testis, and skin should be better studied. Nevertheless, in this picture, the main adverse effects of SARS-CoV-2 on spleen (**A**), bone marrow (**B**), testis (**C**), and skin (**D**) are summarized.

Post-mortem spleens in COVID-19 patients were generally contracted, showing the presence of shrinking capsules. It could be possible to detect mixed thrombi, anemic infarcts, and hemorrhages. Moreover, in several cases, spleens showed atrophic white pulp and relatively enlarged red pulp (Figure 3A). Finally, some of the COVID-19 spleens had a reduced number of CD20-positive B lymphocytes (100%) compared to controls [40].

Hemophagocytosis is a common finding in the bone marrow of subjects who died from severe COVID-19 [63]. Prominent hyperplasia of CD8-positive cytotoxic T cells was randomly found [100]. In particular, the microscopic analysis of bone marrow showed two pathological pictures (Figure 3B): on the one hand, red hematopoietic bone marrow cells were replaced by yellow marrow cells, rich in adipocytes; on the other hand, there was hyperplasia with megakaryocytes [81,82].

#### 3.4.4. SARS-CoV-2 and Kidney

Another organ that was severely affected in cases of SARS-CoV-2 infection is the kidney, showing degenerative changes [90]. Even if the pathological pathway of renal damage in COVID-19 patients is not completely known, the ACE-2 receptor plays a pivotal role in the mechanism of renal infection [117]. Factors that may contribute to acute kidney injury in the presence of SARS-CoV-2 infection are systemic hypoxia, abnormal coagulation, and possible drug-related rhabdomyolysis or hyperventilation [38]. It is important to take into account during the evaluation of the post-mortem findings, any pre-existing comorbidities such as hypertension and diabetes. Indeed, in the evaluation of tissue modification, it is possible to find pre-existing damage not related to COVID-19.

On macroscopic examination, renal signs of shock were observed in many autopsies [100]. Histologically, extensive tubular necrosis mainly in the proximal convoluted tubules was observed; moreover, vascular thrombosis, stasis, and petechial hemorrhages were also described [61,89,91]. Other findings of kidney damage are diffuse proximal tubule injury with the loss of brush border, non-isometric vacuolar degeneration, and necrosis. Occasional hemosiderin granules and pigmented casts were identified [38,116,119].

#### 3.4.5. SARS-CoV-2 and Brain

Different clinical manifestations of the central nervous system (CNS) have been described in COVID-19 patients. These data may be the consequence of the vascular encephalopathy associated with the SARS-CoV-2 infection. The presence of the virus in the brain tissue is not always confirmed: as reported by Matschke et al. [68], SARS-CoV-2 may be detected in the CNS, even if mild neuropathological changes with pronounced neuroinflammation in the brainstem represent the most common finding. Moreover, when it is detected, its presence is strictly related to diffuse inflammatory microvessel endothelial damage, with cytokine production, microencephalopathy, and sometimes microthrombi [129]. In different macroscopic examinations, mild brain swelling and scattered hemorrhagic lesions were described. The histological examination (haematoxylin and eosin stain) could detect intraparenchymal blood foci disrupting the white matter, with the presence of macrophages at the periphery of the lesions [115]. In several post-mortem examinations, cerebral microhemorrhages induced by endotheliitis and vasculopathy have been described [59,64]. Moreover, Al-Dalahmah et al. [118] reported cerebellar hemorrhage and acute infarcts in the dorsal pons and medulla. Other neuropathological findings related to COVID-19 are cerebral cortical infarction with the presence of megakaryocytes and brainstem encephalitis [56]. In contrast, Kantonen et al. [59] did not detect signs of meningitis or encephalitis in any brain area. In the same way, in a recent report performed on 18 subjects who died from/with COVID-19, Solomon et al. [131] reported only hypoxic changes; contrariwise, they did not describe encephalitis or other specific brain changes referable to SARS-CoV-2. In light of these findings, brain damage is not univocal in COVID-19 patients.

#### 3.4.6. SARS-CoV-2 and Liver Tissue

The identification of a specific histopathological pattern of liver damages in COVID-19 patients is very challenging for several reasons. Considering that death occurs in COVID-19 patients with several comorbidities, similarly, liver damage should be analyzed in consideration of the previous clinical status of the deceased subject. Moreover, another important consideration should be made in the analysis of liver alteration after COVID-19: it is related to the drug toxicity during the SARS-CoV-2 infection management that could increase pre-existing liver damage.

Minimal features of inflammation were always detected in the liver samples of COVID-19 patients. During the post-mortem examination, the livers sometimes appeared pale and yellowish, with parenchyma congestion [81]. At the microscopical level, a centrolobular necrosis was detected, associated with discrete lobular or portal inflammation. The main histological changes may be attributed to the hypoxic state related to the SARS-CoV-2 infection at the level of the lung [49,61]. Moreover, histologically, it was possible to identify vascular changes, with the identification of massive lumen dilatation, and partial or complete luminal thrombosis of the portal and sinusoidal vessels. Other findings included fibrotic portal tract signs [77].

#### 3.4.7. SARS-CoV-2 and the Testis

Analyzing the statistical data about COVID-19, fatality rates among men were higher than women, ranging up to 3–5 times in different countries [139]. This data suggested more careful examination in order to ascertain if the male status could be considered an important predictor factor in the COVID-19 outcome. In this way, several groups focused during autopsy on testis findings to show the main modifications that occurred in subjects who died with/from the SARS-CoV-2 infection.

The presence of SARS-CoV-2 in the testis was not always detected. In the post-mortem examination, moderate and severe testis modifications were frequently reported (Figure 3C). Particularly, the number of Leydig cells was reduced compared to the control group. Signs of inflammation were also described, detecting the presence of T lymphocytes and histiocytes [39]. Finally, the presence of microthrombi was sometimes reported both in the testis and in the microvessels of the prostate [111].

#### 3.4.8. SARS-CoV-2 and Skin Tissue

The ACE-2 receptor may be considered the entrance door for SARS-CoV-2; considering that it may be considered ubiquitous, SARS-CoV-2 could be potentially detected in all organs and tissues. Liu et al. [41] focused their study on the sweat glands and sweat ducts, and on small blood vessels in the skin. Based on their results, SARS-CoV-2 was also detected at this level, generating a vasculitis with prominent infiltration of lymphocytes and enlarged vascular endothelial cells (Figure 3D).

Occidental et al. [124] focused on the superficial dermis, showing the presence of thrombotic microangiopathy. Moreover, they described lesions such as vesicle formation. These findings suggest that in several cases, the lesion on the dermis caused by SARS-CoV-2 infection may be similar to bullous diseases, underlying a role for the immune response.

## 4. Discussion

Despite advanced technologies in the diagnostic fields, to date, autopsy remains the gold standard method to understand the biological features and the pathogenesis of unknown infections [65,66], especially when awareness of a pathogen is restricted and the impact on the healthcare system is substantial. Ideally, the knowledge gained through this technique may positively influence therapeutic strategies, ultimately reducing mortality. However, the questionable choice of several countries to limit the post-mortem investigation has severely limited the practice of autopsies of COVID-19 patients [10]. Indeed, in the first pandemic months, many governments—including the Italian government—discouraged autopsy procedures in COVID-19 deaths [136], limiting its practice to forensic cases. During the same period, many national and international guidelines suggested safety procedures to perform post-mortem examinations for those people who died with COVID-19 in a safe manner [7,8,9]. Subsequently, as discussed in the present review, many autopsy studies have been published.

SARS-CoV spread in 2002/2003 and is an acute viral pulmonary disease that requires, in critical cases, intensive care and especially mechanical ventilation [140]. The post-mortem investigations were fundamental in order to define the histopathological pattern. Particularly, DAD with the presence of hyaline membranes, hyperplasia of type 2 pneumocytes, and distal arterial thrombi were observed [141].

In the case of MERS-CoV, in an effort to improve the knowledge about this new pathogen, the scientific community focused mainly on small animal models [142]. This is demonstrated by the limited number of autopsies, even if the mortality rate is higher compared to the other coronaviruses. It is important to note that the spread of MERS is very limited compared to SARS-CoV-2, considering that the case number is limited to about 2500, with a higher mortality rate (about 35% of cases) [30]. The limited number of cases, combined with the diffusion in Middle East countries, may be considered two important factors in the evaluation of the lack of post-mortem investigations, particularly compared to the post-mortem investigations performed during the pandemic infection of SARS-CoV-2.

Responding to a different alarming paper of the scientific community [11,143], in consideration of the increase of subjects who died with/from COVID-19, the number of post-mortem investigations is considerably increased in the second phase of the pandemic. As demonstrated in this review, although it was initially thought that SARS-CoV-2 affects only respiratory systems, thanks to post-mortem investigations, it was ascertained that this infection may cause different damage on other organs such as the heart, kidneys, and liver. Moreover, to date, considering the recrudescence of COVID-19, there is an immediate necessity to investigate all involvement, for example, clarifying the involvement of the digestive system [23]. These data will be very useful in the management of COVID-19 survivors.

In this way, evaluating the necessity to further improve the knowledge about COVID-19, a number of clinical organizations have announced their interest in collecting and analyzing data from patients with COVID-19. Moreover, several large-scale prospective data collections are ongoing, such as the LEOSS registry (Lean European Open Survey on SARS-CoV-2-Infected Patients) or the CAPACITYCOVID registry (registry of patients with COVID-19 including cardiovascular risk and complications) [144]. An interesting collaborative study was published by Borczuk et al. [133]. The authors collected the data of 68 autopsies from Italy and New York City, and their findings show a high complexity of the COVID-19 disease; for example, they frequently describe a high frequency of thrombi, suggesting an impact on clinical management. It is fundamental to underline the usefulness of mobilizing and harmonizing basic and applied research worldwide. Furthermore, when a patient dies unexpectedly at home, perhaps after a short period of flu-like symptoms, without hospitalization and/or nasopharyngeal swab investigation, the post-mortem investigation is fundamental in order to define if these unknown fatal cases may be due to COVID-19 [80,145].

A crucial aspect of the post-mortem investigation is the safety of the personnel involved. To date, no evidence of the infection during the post-mortem investigation has been supplied. In this way, we firmly believe that when an autopsy is performed in a proper biosafety autopsy room using personal protective equipment [9,146], as recommended by the different international guidelines, the complete post-mortem investigation is a safe procedure, even if several recent articles discussed the persistence of the SARS-CoV-2 virus in the post-mortem period [147]. Really, they did not demonstrate the vitality of the virus; however, they only detected the positivity of swab samples collected during autopsy procedures through real-time PCR (polymerase chain reaction). For this reason, it is fundamental to remark that before sending alarming massages, the forensic community is called on to fully comprehend the weight of the evidence. It is important to highlight that the lessons learned from this review urge all the personnel involved in autopsies (technicians, biologists, pathologists) to take into account the presence of SARS-CoV-2 in all suspected deaths during the pandemic, wearing appropriate personal protective equipment adopting the suggested procedure to perform autopsy safely [53,148]. At the same time, the value of the autopsy in the management of unknown diseases is indisputable.

For over two centuries, autopsy has been considered a fundamental diagnostic technique, particularly in cases of new or little-known human disorders. However, today, it is often treated as obsolete. This is perhaps one of the explanations for the fact that in the first phase of the pandemic, autopsies were performed only in a few cases, with delays and often discouraged, if not even prohibited, by more than one country [149].

In conclusion, considering that SARS-CoV and MERS-CoV have had different characteristics both from the clinical and diffusion viewpoints, SARS-CoV-2 seems to have unique characteristics that we will only understand as the epidemic evolves, taking into account the evidence for host-dependent RNA editing in the transcriptome of SARS-CoV-2 [150].

In light of these considerations, autopsies during the COVID-19 infection should not be considered as an exception, but rather as a mandatory tool for the management of the pandemic disease. Forensic medicine could offer the correct answer to this challenge, determining the exact cause of death, contributing to the reliability of death statistics, comprehending the pathological mechanisms of SARS-CoV-2, mapping the presence of the virus after death, as well as addressing disputes on medico-legal issues [151].

The so-called “take-home message” of this literature review is that in the near future, in the case of a new pandemic pathogen, the lesson “to learn from the dead” [152] should be considered a rule and not only an opportunity, as the scientific advantage to be obtained from the experience of autopsies is incommensurable both from research and public health viewpoints.

## Figures and Tables

**Table 1:** General data obtained analyzing the studies included in this literature review matching the keywords “Autopsy” OR “Post-mortem” AND “SARS-CoV” (severe acute respiratory syndrome coronavirus).

Source	Country	Autopsy (Number and Sex)	Kind of Autopsy	Diagnostic Technique for SARS-CoV
Farcas et al. and Oudit et al. [16,17]	Canada	21 (10 Female (F); 11 Male (M))	Complete autopsies	RT-PCR (Reverse transcriptase-polymerase chain reaction)
Mazzulli et al. [18]	Canada	11 (5 F; 6 M)	Complete autopsies focusing on the lung	RT-PCR
	America	32		
He et al. [19]	China	4 (not available)	Complete autopsies	Not available
Zhang et al. [20]	China	1 (not available)	Complete autopsy	RT-PCR
Nicholls et al. [21]	China	6 (2 F; 4 M)	1 full autopsy; 5 lung investigations	RT-PCR
Pei et al. [22]	China	6 (not available)	Complete autopsies	RT-PCR
Shi et al. [23]	China	7 (1 F; 6 M)	Complete autopsies	RT-PCR
He et al. [24]	China	4 (not available)	Complete autopsies	RT-PCR
Tse et al. [25]	Hong Kong	7 (1 F; 6 M)	6 full autopsies (excluding the brain); 1 limited autopsy (lung and heart)	RT-PCR
Franks et al. [26]	Singapore	8 (2 F; 6 M)	Complete autopsies focusing on the lung	7/8 RT-PCR
Kong et al. [27]	Singapore	7 (2 F; 5 M)	Lung tissues	RT-PCR
Hsiao et al. [28]	Taiwan	4 (not available)	Limited autopsies (lung tissue)	RT-PCR
Hsiao et al. [29]	Taiwan	1 F	Complete autopsy	RT-PCR
	ASIA	55		

**Table 2:** General data obtained analyzing the studies included in this literature review matching the keywords “Autopsy” OR “Post-mortem” AND “MERS” (Middle East Respiratory Syndrome).

Source	Country	Autopsy (Number and Sex)	Kind of Autopsy	Diagnosis of MERS-CoV
Ng et al. [33]	United Arab Emirates	1 M	Complete autopsy	RT-PCR
Alsaad et al. [34]	Saudi Arabia	1 M	Tissue needle biopsies were obtained from brain, heart, lung, liver, kidney, and skeletal muscles	RT-PCR

**Table 3:** General data obtained analyzing the studies included in this literature review matching the keywords “Autopsy” OR “Post-mortem” AND “SARS-CoV-2” (severe acute respiratory syndrome coronavirus 2) OR “COVID-19” (coronavirus disease 2019).

Source	Country	Autopsy (Number and Sex)	Kind of Autopsy	Diagnosis of SARS-CoV-2
Wang et al. [36]	China	2 (1 F; 1 M)	Complete autopsy	RT-PCR
Li et al. [37]	China	30 (10 F; 20 M)	Minimally invasive autopsies	RT-PCR
Su et al. [38]	China	26 (7 F; 19 M)	Complete autopsies, focusing on the kidney	RT-PCR
Yang et al. [39]	China	12 M	Complete autopsies, focusing on the testis	RT-PCR
Liu et al. [40]	China	12 (5 F; 7 M)	Complete autopsy	RT-PCR
Lui et al. [41]	China	5 (3 F; 2 M)	Complete autopsies, focusing on the skin	RT-PCR
Tian et al. [42]	China	2 (1 F; 1 M)	Lung analysis	RT-PCR
Yao et al. [43]	China	3 M	Complete autopsy	RT-PCR
Karami et al. [44]	Iran	1 F	Complete autopsy	RT-PCR
Okudela et al. [45]	Japan	1 F	Complete autopsy	RT-PCR
Adachi et al. [46]	Japan	1 F	Complete autopsy (with the exception of the brain and bone marrow)	RT-PCR
	ASIA	95		
Lax et al. [47]	Austria	11 (3 F; 8 M)	Complete autopsies	RT-PCR
Grosse et al. [48]	Austria	14 (5 F; 9 M)	Complete autopsies	RT-PCR
Schmit et al. [49]	Belgium	14 (4 F; 10 M)	Complete autopsies	RT-PCR
Remmelink et al. [50]	Belgium	17 (5 F; 12 M)	Complete autopsies	RT-PCR
Jacobs et al. [51]	Belgium	1 M	Complete autopsy	RT-PCR
Radermecker et al [52]	Belgium	4 (1 F; 3 M)	Complete autopsies	RT-PCR
Puzovic et al. [53]	Croatia	1 M	Complete autopsy	RT-PCR
Schwensen et al. [54]	Denmark	1 F	Complete autopsy	RT-PCR
Leth et al. [55]	Denmark	1 M	Complete autopsy	RT-PCR
Jensen et al. [56]	England	2 M	Complete autopsies, focusing on the brain	RT-PCR
Youd and Moore [57]	England	9 (5 F; 4 M)	Complete autopsies	3 confirmed cases with RT-PCR
Griffin [58]	England	1 F	Complete autopsy	RT-PCR
Kantonen et al. [59]	Finland	4 (1 F; 3 M)	Complete autopsies	RT-PCR
Ducloyer et al. [60]	France	1 M	Complete autopsy	RT-PCR
Gauchotte et al. [61]	France	1 M	Complete autopsy	RT-PCR (Post-mortem samples)
Ackermann et al. [62]	Germany	7 (2 F; 5 M)	Complete autopsies, focusing on lung samples	Not described
Swoboda et al. [63]	Germany	15 (15 M)	Complete autopsies, focusing on bone marrow specimens	RT-PCR
Kirschenbaum et al. [64]	Germany	4 (1 F; 3 M)	Complete autopsies, focusing on brain tissue	RT-PCR
Fitzek et al. [65]	Germany	1 M	Complete autopsy	RT-PCR
Wichmann et al. [66]	Germany	12 (3 F; 9 M)	Complete autopsies	RT-PCR
Edler et al. [67] and Matschke et al. [68]	Germany	80 (34 F; 46 M)	Complete autopsies	RT-PCR
Heinrich et al. [69]	Germany	1 M	Complete autopsy	RT-PCR
Dettmeyer et al. [70]	Germany	3 M	Complete autopsy	RT-PCR
Klein et al. [71]	Germany	8 (4 F; 4 M)	Complete autopsy	RT-PCR
Schaller et al. [72]	Germany	12 (5 F; 7 M)	Complete autopsies	RT-PCR
Lindner et al. [73]	Germany	39 (23 F; 16 M)	Complete autopsies, focusing on the heart	RT-PCR
Wagner et al. [74]	Germany	2 M	Complete autopsy	RT-PCR
Bösmüller et al. [75]	Germany	4 (1 F; 3 M)	Complete autopsies	RT-PCR
Bulfamante et al. [76]	Italy	6 (1 F; 4 M)	Complete autopsies	RT-PCR
Sonzogni et al [77]	Italy	48 (8 F; 22 M)	30 partial autopsies limited to lungs, heart, and liver, 18 complete autopsies, excluding the brain	RT-PCR
Carsana et al. [78]	Italy	38 (5 F; 33 M)	Complete autopsies, focusing on lung samples	RT-PCR
Cipolloni et al. [79]	Italy	2 (2 M)	Complete autopsies	1 RT-PCR, 1 Immunohistochemistry
Tombolini et al. [80]	Italy	2 F	Complete autopsies	RT-PCR
Falasca et al. [81]	Italy	22 (7 F; 15 M)	Complete autopsies, excluding the brain	RT-PCR
Roncati et al. [82]	Italy	1 M	Complete autopsy	RT-PCR
Bussani et al. [83]	Italy	41 (16 F; 25 M)	Complete autopsies	RT-PCR
Damiani et al. [84]	Italy	9 (2 F; 7 M)	Complete autopsies	RT-PCR
Titi et al. [85]	Italy	1 M	Complete autopsy	RT-PCR
Del Nonno et al. [86]	Italy	1 W	Complete autopsy	RT-PCR with negativization
Basso et al. [87]	USA & Italy	21 (6 F; 15 M)	Complete autopsies, focusing on the heart	RT-PCR
Chmielik et al. [88]	Poland	3 M	Complete autopsies	RT-PCR
Cirstea et al. [89]	Romania	1 F	Complete autopsy	RT-PCR
Popa et al. [90]	Romania	1 M	Complete autopsy	RT-PCR
Oprinca et al. [91]	Romania	3 (1 F; 2 M)	Complete autopsies	RT-PCR
Kogan et al. [92]	Russia	4 (not available)	Complete autopsies, focusing on heart tissues	RT-PCR
Kovylina et al. [93]	Russia	37 (not available)	Partial autopsies with analysis of the lung and kidney	RT-PCR
Navarro Conte et al. [94]	Spain	1 M	Complete autopsy	---
Yan et al. [95]	Spain	1 F	Complete autopsy	RT-PCR
Varela Barca et al. [96]	Spain	1 W	Complete autopsy	Immunohistochemistry for SARS-Cov-2
Hellman et al. [97]	Sweden	2 M	Complete autopsies	RT-PCR
Suesse et al. [98]	Switzerland	1 M	Complete autopsy	RT-PCR
Aguiar et al. [99]	Switzerland	1 W	Complete autopsy	RT-PCR
Menter et al. [100]	Switzerland	21 (4 F; 17 M)	17 Complete autopsies, 4 partial autopsies	RT-PCR
Nienhold et al. [101]	Switzerland	16 (3 F; 13 M)	Complete autopsies	RT-PCR
Varga et al. [102]	Switzerland	3 (1 F; 2 M)	Complete autopsies	RT-PCR
Schweitzer et al. [103]	Switzerland	1 M	Complete autopsy	RT-PCR
Seetulsingh et al. [104]	United Kingdom	1 M	Complete autopsy	RT-PCR on lung tissue
	EUROPE	580		
Dolhnikoff et al. [105]	Brazil	1 M	Complete autopsy	RT-PCR
Duarte-Neto et al. [106]	Brazil	10 (5 F; 5 M)	Complete autopsies	RT-PCR
Santana et al. [107]	Brazil	1 M	Complete autopsy	RT-PCR
Miggiolaro et al. [108]	Brazil	2 (1 F; 1 M)	Two lung biopsies	RT-PCR
Gonzalez et al. [109]	Cuba	10 (4 F; 6 M)	Partial autopsies	RT-PCR
Barton et al. [110]	USA (OK)	2 (M)	Complete autopsies	RT-PCR
Elsoukkary et al. [111]	USA	32 (10 F; 22 M)	28/32 complete autopsies (excluding the brain); 4/32 lung examination	31/32 RT-PCR
Craver et al. [112]	USA	1 M	Complete autopsy	RT-PCR
Fox et al. [113]	USA	10 (not available)	Complete autopsies, focusing on the lung	RT-PCR
Prilutskiy et al. [114]	USA	4 (1 F; 3 M)	Complete autopsy	RT-PCR
Reichard et al. [115]	USA	1 M	Complete autopsy, focusing on the brain	RT-PCR
Bradley et al. [116]	USA	14 (8 F; 6 M)	7 complete autopsies, 7 in-situ dissections	RT-PCR
Farkash et al. [117]	USA	1 M	Complete autopsy	RT-PCR
Al-Dalahmah et al. [118]	USA	1 M	Complete autopsy	RT-PCR
Santoriello et al. [119]	USA	42 (13 F; 29 M)	Complete autopsies, focusing on kidney	RT-PCR
Grimes et al. [120]	USA	2 M	Complete autopsy	RT-PCR
Buja et al. [121]	USA	3 M	Complete autopsies, excluding the brain	RT-PCR
Barna et al. [122]	USA	3 (1 F; 2 M)	Complete autopsies	RT-PCR
Sauter et al. [123]	USA	8 (4 F; 4 M)	Complete autopsies	RT-PCR
Occidental et al. [124]	USA	4 M	Complete autopsies, focusing on skin lesions	RT-PCR
Iuga et al. [125]	USA (NY)	5 (1 F; 4 M)	Complete autopsies	RT-PCR
Konopka et al. [126]	USA (MI)	1	Complete autopsy	RT-PCR
Lacy et al [127]	USA (MI)	1	Complete autopsy	RT-PCR
Paniz Mondolfi et al. [128]	USA (NY)	1 (M)	Complete autopsy	RT-PCR
Nuovo et al. [129]	USA	13 (6 F; 7 M)	Brain autopsies	RT-PCR
Rapkiewicz et al. [130]	USA	7 (4 F; 3 M)	Complete autopsies	RT-PCR
Solomon et al. [131]	USA	21 (4 F; 14 M)	Complete autopsies, focusing on the brain	RT-PCR
Magro et al. [132]	USA	2 M	Complete autopsies	RT-PCR
Borczuk et al. [133]	USA & Italy	68 (48 USA; 20 Italy); (21 F; 47 M)	Complete autopsies, focusing on the lung	RT-PCR
	AMERICA	251		
Attoh et al. [134]	Ghana	3 M	Complete autopsies	2/3 RT-PCR
Khaba et al. [135]	South Africa	1 M	Complete autopsy	RT-PCR
	AFRICA	4		

## Data Availability

All data are included in the present publication.

## References

[1] Guarner J. (2020). Three Emerging Coronaviruses in Two Decades: The Story of SARS, MERS, and Now COVID-19. Am. J. Clin. Pathol..

[2] Cheng V.C.C., Lau S.K.P., Woo P.C.Y., Yuen K.Y. (2007). Severe Acute Respiratory Syndrome Coronavirus as an Agent of Emerging and Reemerging Infection. Clin. Microbiol. Rev..

[3] CDC (2005). Severe Acute Respiratory Syndrome (SARS). https://www.cdc.gov/sars/index.html.

[4] Hui D.S., Azhar E.I., Kim Y.-J., Memish Z.A., Oh M.-D., Zumla A. (2018). Middle East respiratory syndrome coronavirus: Risk factors and determinants of primary, household, and nosocomial transmission. Lancet Infect. Dis..

[5] WHO (2020). Middle East Respiratory Syndrome Coronavirus (MERS-CoV)—United Arab Emirates. https://www.who.int/csr/don/31-january-2020-mers-united-arab-emirates/en/.

[6] Lescure F.-X., Bouadma L., Nguyen D., Parisey M., Wicky P.-H., Behillil S., Gaymard A., Bouscambert-Duchamp M., Donati F., Le Hingrat Q. (2020). Clinical and virological data of the first cases of COVID-19 in Europe: A case series. Lancet Infect. Dis..

[7] Centers for Disease Control and Prevention (CDC) Collection and Submission of Postmortem Specimens from Deceased Persons with Known or Suspected. Ad Interim Guid 2020. https://www.cdc.gov/coronavirus/2019-ncov/hcp/guidance-postmortem-specimens.html.

[8] Hanley B., Lucas S.B., Youd E., Swift B., Osborn M. (2020). Autopsy in suspected COVID-19 cases. J. Clin. Pathol..

[9] WHO Interm Guidance Infection Prevention and Control for the Safe Management of a Dead Body in the Context of COVID-19. https://www.who.int/publications/i/item/infection-prevention-and-control-for-the-safe-management-of-a-dead-body-in-the-context-of-covid-19-interim-guidance..

[10] Salerno M., Sessa F., Piscopo A., Montana A., Torrisi M., Patanè F., Murabito P., Volti G.L., Pomara C. (2020). No Autopsies on COVID-19 Deaths: A Missed Opportunity and the Lockdown of Science. J. Clin. Med..

[11] Pomara C., Volti G.L., Cappello F. (2020). COVID-19 Deaths: Are We Sure It Is Pneumonia? Please, Autopsy, Autopsy, Autopsy!. J. Clin. Med..

[12] D’Errico S., Zanon M., Montanaro M., Radaelli D., Sessa F., Di Mizio G., Montana A., Corrao S., Salerno M., Pomara C. (2020). More than Pneumonia: Distinctive Features of SARS-Cov-2 Infection. From Autopsy Findings to Clinical Implications: A Systematic Review. Microorganisms.

[13] Calabrese F., Pezzuto F., Fortarezza F., Hofman P., Kern I., Panizo A., Von Der Thüsen J., Timofeev S., Gorkiewicz G., Lunardi F. (2020). Pulmonary pathology and COVID-19: Lessons from autopsy. The experience of European Pulmonary Pathologists. Virchows Arch..

[14] Al Nemer A. (2020). Histopathologic and Autopsy Findings in Patients Diagnosed With Coronavirus Disease 2019 (COVID-19): What We Know So Far Based on Correlation With Clinical, Morphologic and Pathobiological Aspects. Adv. Anat. Pathol..

[15] Roncati L., Lusenti B. (2020). The «moonlighting protein» able to explain the Th1 immune lockdown in severe COVID-19. Med. Hypotheses.

[16] Farcas G.A., Poutanen S.M., Mazzulli T., Willey B.M., Butany J., Asa S.L., Faure P., Akhavan P., Low D.E., Kain K.C. (2005). Fatal Severe Acute Respiratory Syndrome Is Associated with Multiorgan Involvement by Coronavirus. J. Infect. Dis..

[17] Oudit G.Y., Kassiri Z., Jiang C., Liu P.P., Poutanen S.M., Penninger J.M., Butany J. (2009). SARS-coronavirus modulation of myocardial ACE2 expression and inflammation in patients with SARS. Eur. J. Clin. Investig..

[18] Mazzulli T., Farcas G.A., Poutanen S.M., Willey B.M., Low D.E., Butany J., Asa S.L., Kain K.C. (2004). Severe Acute Respiratory Syndrome–associated Coronavirus in Lung Tissue. Emerg. Infect. Dis..

[19] He L., Ding Y.Q., Che X.Y., Zhang Q.L., Huang Z.X., Wang H.J. (2003). Expression of the monoclonal antibody against nucleocapsid antigen of SARS-associated coronavirus in autopsy tissues from SARS patients. Di Yi Jun Yi Da Xue Xue Bao.

[20] Zhang Q.L., Ding Y.Q., Hou J.L., He L., Huang Z.X., Wang H.J. (2003). Detection of severe acute respiratory syndrome (SARS)-associated coronavirus RNA in autopsy tissues with in situ hybridization. Di Yi Jun Yi Da Xue Xue Bao.

[21] Nicholls J.M., Poon L.L., Lee K.C., Ng W.F., Lai S.T., Leung C.Y., Chu C.M., Hui P.K., Mak K.L., Lim W. (2003). Lung pathology of fatal severe acute respiratory syndrome. Lancet.

[22] Pei F., Zheng J., Gao Z.F., Zhong Y.F., Fang W.G., Gong E.C. (2005). Lung pathology and pathogenesis of severe acute respiratory syndrome: A report of six full autopsies. Zhonghua Bing Li Xue Za Zhi.

[23] Shi X., Gong E., Gao D., Zhang B., Zheng J., Gao Z., Zhong Y., Zou W., Wu B., Fang W. (2005). Severe Acute Respiratory Syndrome Associated Coronavirus Is Detected in Intestinal Tissues of Fatal Cases. Am. J. Gastroenterol..

[24] He L., Ding Y., Zhang Q., Che X., He Y., Shen H., Wang H., Li Z., Zhao L., Geng J. (2006). Expression of elevated levels of pro-inflammatory cytokines in SARS-CoV-infected ACE2+cells in SARS patients: Relation to the acute lung injury and pathogenesis of SARS. J. Pathol..

[25] Tse G.M.-K., To K.-F., Chan P.K.-S., Lo A.W.I., Ng K.-C., Wu A., Lee N., Wong H.-C., Mak S.-M., Chan K.-F. (2004). Pulmonary pathological features in coronavirus associated severe acute respiratory syndrome (SARS). J. Clin. Pathol..

[26] Franks T.J., Chong P.Y., Chui P., Galvin J.R., Lourens R.M., Reid A.H., Selbs E., Mcevoy C.P.L., Hayden C.D.L., Fukuoka J. (2003). Lung pathology of severe acute respiratory syndrome (SARS): A study of 8 autopsy cases from Singapore. Hum. Pathol..

[27] Kong S.L., Chui P., Lim B., Salto-Tellez M. (2009). Elucidating the molecular physiopathology of acute respiratory distress syndrome in severe acute respiratory syndrome patients. Virus Res..

[28] Hsiao C.H., Wu M.-Z., Chen C.-L., Hsueh P.-R., Hsieh S.-W., Yang P.-C., Su I.-J. (2005). Evolution of pulmonary pathology in severe acute respiratory syndrome. J. Formos. Med Assoc..

[29] Hsiao C.-H., Wu M.-Z., Hsieh S.-W., Chien L.-C., Hwang K.-C., Su I.-J. (2004). Clinicopathology of severe acute respiratory syndrome: An autopsy case report. J. Formos. Med Assoc..

[30] Da Costa V.G., Moreli M.L., Saivish M.V. (2020). The emergence of SARS, MERS and novel SARS-2 coronaviruses in the 21st century. Arch. Virol..

[31] Meyerholz D.K., Lambertz A.M., McCray P.B. (2016). Dipeptidyl Peptidase 4 Distribution in the Human Respiratory Tract Implications for the Middle East Respiratory Syndrome. Am. J. Pathol..

[32] Baseler L.J., Falzarano D., Scott D.P., Rosenke R., Thomas T., Munster V.J., Feldmann H., de Wit E. (2016). An Acute Immune Response to Middle East Respiratory Syndrome Coronavirus Replication Contributes to Viral Pathogenicity. Am. J. Pathol..

[33] Ng D.L., Al Hosani F., Keating M.K., Gerber S.I., Jones T.L., Metcalfe M.G., Tong S., Tao Y., Alami N.N., Haynes L.M. (2016). Clinicopathologic, Immunohistochemical, and Ultrastructural Findings of a Fatal Case of Middle East Respiratory Syndrome Coronavirus Infection in the United Arab Emirates, April 2014. Am. J. Pathol..

[34] Alsaad K.O., Hajeer A.H., Al Balwi M., Al Moaiqel M., Al Oudah N., Al Ajlan A., Aljohani S., Alsolamy S., E Gmati G., Balkhy H. (2018). Histopathology of Middle East respiratory syndrome coronovirus (MERS-CoV) infection—Clinicopathological and ultrastructural study. Histopathology.

[35] Szabolcs M., Sauter J.L., Frosina D., Geronimo J.A., Hernandez E., Selbs E., Rapkiewicz A.V., Rekhtman N., Baine M.K., Jäger E. (2021). Identification of Immunohistochemical Reagents for In Situ Protein Expression Analysis of Coronavirus-associated Changes in Human Tissues. Appl. Immunohistochem. Mol. Morphol..

[36] Wang C., Xie J., Zhao L., Fei X., Zhang H., Tan Y., Nie X., Zhou L., Liu Z., Ren Y. (2020). Alveolar macrophage dysfunction and cytokine storm in the pathogenesis of two severe COVID-19 patients. EBioMedicine.

[37] Li Y., Wu J., Wang S., Li X., Zhou J., Huang B., Luo D., Cao Q., Chen Y., Chen S. (2021). Progression to fibrosing diffuse alveolar damage in a series of 30 minimally invasive autopsies with COVID-19 pneumonia in Wuhan, China. Histopathology.

[38] Su H., Yang M., Wan C., Yi L.-X., Tang F., Zhu H.-Y., Yi F., Yang H.-C., Fogo A.B., Nie X. (2020). Renal histopathological analysis of 26 postmortem findings of patients with COVID-19 in China. Kidney Int..

[39] Yang M., Chen S., Huang B., Zhong J.-M., Su H., Chen Y.-J., Cao Q., Ma L., He J., Li X.-F. (2020). Pathological Findings in the Testes of COVID-19 Patients: Clinical Implications. Eur. Urol. Focus.

[40] Liu Q., Shi Y., Cai J., Duan Y., Wang R., Zhang H., Ruan Q., Li J., Zhao L., Ping Y. (2020). Pathological changes in the lungs and lymphatic organs of 12 COVID-19 autopsy cases. Natl. Sci. Rev..

[41] Liu J., Li Y., Liu L., Hu X., Wang X., Hu H., Hu Z., Zhou Y., Wang M. (2020). Infection of human sweat glands by SARS-CoV-2. Cell Discov..

[42] Tian S., Hu W., Niu L., Liu H., Xu H., Xiao S.-Y. (2020). Pulmonary Pathology of Early-Phase 2019 Novel Coronavirus (COVID-19) Pneumonia in Two Patients with Lung Cancer. J. Thorac. Oncol..

[43] Yao X.H., Li T.Y., He Z.C., Ping Y.F., Liu H.W., Yu S.C., Mou H.M., Wang L.H., Zhang H.R., Fu W.J. (2020). A pathological report of three COVID-19 cases by minimally invasive autopsies. Zhonghua Bing Li Xue Za Zhi Chin. J. Pathol..

[44] Karami P., Naghavi M., Feyzi A., Aghamohammadi M., Novin M.S., Mobaien A., Qorbanisani M., Karami A., Norooznezhad A.H. (2020). WITHDRAWN: Mortality of a pregnant patient diagnosed with COVID-19: A case report with clinical, radiological, and histopathological findings. Travel Med. Infect. Dis..

[45] Okudela K., Hayashi H., Yoshimura Y., Sasaki H., Horiuchi H., Miyata N., Tachikawa N., Tsuchiya Y., Mitsui H., Ohashi K. (2020). A Japanese case of COVID-19: An autopsy report. Pathol. Int..

[46] Adachi T., Chong J.-M., Nakajima N., Sano M., Yamazaki J., Miyamoto I., Nishioka H., Akita H., Sato Y., Kataoka M. (2020). Clinicopathologic and Immunohistochemical Findings from Autopsy of Patient with COVID-19, Japan. Emerg. Infect. Dis..

[47] Lax S.F., Skok K., Zechner P., Kessler H.H., Kaufmann N., Koelblinger C., Vander K., Bargfrieder U., Trauner M. (2020). Pulmonary Arterial Thrombosis in COVID-19 With Fatal Outcome: Results from a Prospective, Single-Center, Clinicopathologic Case Series. Ann. Intern. Med..

[48] Grosse C., Grosse A., Salzer H.J., Dünser M.W., Motz R., Langer R. (2020). Analysis of cardiopulmonary findings in COVID-19 fatalities: High incidence of pulmonary artery thrombi and acute suppurative bronchopneumonia. Cardiovasc. Pathol..

[49] Schmit G., Lelotte J., Vanhaebost J., Horsmans Y., Van Bockstal M., Baldin P. (2020). The Liver in COVID-19-Related Death: Protagonist or Innocent Bystander?. Pathobiology.

[50] Remmelink M., De Mendonça R., D’Haene N., De Clercq S., Verocq C., Lebrun L., Lavis P., Racu M.-L., Trépant A.-L., Maris C. (2020). Unspecific post-mortem findings despite multiorgan 1 viral spread in COVID-19 patients. Crit. Care.

[51] Jacobs W., Lammens M., Kerckhofs A., Voets E., Van San E., Van Coillie S., Peleman C., Mergeay M., Sirimsi S., Matheeussen V. (2020). Fatal lymphocytic cardiac damage in coronavirus disease 2019 (COVID-19): Autopsy reveals a ferroptosis signature. ESC Hear. Fail..

[52] Radermecker C., Detrembleur N., Guiot J., Cavalier E., Henket M., D’Emal C., Vanwinge C., Cataldo D., Oury C., Delvenne P. (2020). Neutrophil extracellular traps infiltrate the lung airway, interstitial, and vascular compartments in severe COVID-19. J. Exp. Med..

[53] Puzović V., Baković M., Bubalo P., Mayer D. (2020). Accidental death from a fall from height at workplace turned out to be a COVID-19 death. Forensic Sci. Int. Rep..

[54] Schwensen H.F., Borreschmidt L.K., Storgaard M., Redsted S., Christensen S., Madsen L.B. (2020). Fatal pulmonary fibrosis: A post-COVID-19 autopsy case. J. Clin. Pathol..

[55] Leth P.M., Rasmussen C.H., Pagh M. (2020). Findings in post-mortem CT and autopsy in a 53-year-old-man with COVID-19. Ugeskr. Laeger..

[56] Jensen M.P., Le Quesne J., Officer-Jones L., Teodòsio A., Thaventhiran J., Ficken C., Goddard M., Smith C., Menon D., Allinson K.S. (2020). Neuropathological findings in two patients with fatal COVID-19. Neuropathol. Appl. Neurobiol..

[57] Youd E., Moore L. (2020). COVID-19 autopsy in people who died in community settings: The first series. J. Clin. Pathol..

[58] Griffin K.J. (2021). Autopsy in the time of COVID. Diagn. Histopathol..

[59] Kantonen J., Mahzabin S., Mäyränpää M.I., Tynninen O., Paetau A., Andersson N., Sajantila A., Vapalahti O., Carpén O., Kekäläinen E. (2020). Neuropathologic features of four autopsied COVID-19 patients. Brain Pathol..

[60] Ducloyer M., Gaborit B., Toquet C., Castain L., Bal A., Arrigoni P.P., LeComte R., Clement R., Sagan C. (2020). Complete post-mortem data in a fatal case of COVID-19: Clinical, radiological and pathological correlations. Int. J. Leg. Med..

[61] Gauchotte G., Venard V., Segondy M., Cadoz C., Esposito-Fava A., Barraud D., Louis G. (2021). SARS-Cov-2 fulminant myocarditis: An autopsy and histopathological case study. Int. J. Leg. Med..

[62] Ackermann M., Verleden S.E., Kuehnel M., Haverich A., Welte T., Laenger F., Vanstapel A., Werlein C., Stark H., Tzankov A. (2020). Pulmonary Vascular Endothelialitis, Thrombosis, and Angiogenesis in Covid-19. N. Engl. J. Med..

[63] Swoboda J., Wittschieber D., Sanft J., Kleemann S., Elschner S., Ihle H., Hubig M., Pletz M.W., Mall G., Gassler N. (2021). Bone marrow haemophagocytosis indicates severe infection with severe acute respiratory syndrome coronavirus 2. Histopathology.

[64] Kirschenbaum D., Imbach L.L., Rushing E.J., Frauenknecht K.B.M., Gascho D., Ineichen B.V., Keller E., Kohler S., Lichtblau M., Reimann R.R. (2020). Intracerebral endotheliitis and microbleeds are neuropathological features of COVID-19. Neuropathol. Appl. Neurobiol..

[65] Fitzek A., Sperhake J., Edler C., Schröder A.S., Heinemann A., Heinrich F., Ron A., Mushumba H., Lütgehetmann M., Püschel K. (2020). Evidence for systematic autopsies in COVID-19 positive deceased: Case report of the first German investigated COVID-19 death. Rechtsmedizin.

[66] Wichmann D., Sperhake J.-P., Lütgehetmann M., Steurer S., Edler C., Heinemann A., Heinrich F., Mushumba H., Kniep I., Schröder A.S. (2020). Autopsy Findings and Venous Thromboembolism in Patients With COVID-19: A prospective cohort study. Ann. Intern. Med..

[67] Edler C., Schröder A.S., Aepfelbacher M., Fitzek A., Heinemann A., Heinrich F., Klein A., Langenwalder F., Lütgehetmann M., Meißner K. (2020). Dying with SARS-CoV-2 infection—an autopsy study of the first consecutive 80 cases in Hamburg, Germany. Int. J. Leg. Med..

[68] Matschke J., Lütgehetmann M., Hagel C., Sperhake J.P., Schröder A.S., Edler C., Mushumba H., Fitzek A., Allweiss L., Dandri M. (2020). Neuropathology of patients with COVID-19 in Germany: A post-mortem case series. Lancet Neurol..

[69] Heinrich F., Sperhake J.-P., Heinemann A., Mushumba H., Lennartz M., Nörz D., Glatzel M., Lütgehetmann M., Püschel K. (2020). Germany’s first COVID-19 deceased: A 59-year-old man presenting with diffuse alveolar damage due to SARS-CoV-2 infection. Virchows Arch..

[70] Dettmeyer R., Lasczkowski G., Weber A., Wolter T., Kernbach-Wighton G. (2020). Histopathological findings following SARS-CoV-2 infection with and without treatment—Report of three autopsies. Rechtsmedizin.

[71] Klein A., Edler C., Fitzek A., Fröb D., Heinemann A., Meißner K., Mushumba H., Püschel K., Schröder A.S., Sperhake J.P. (2020). The first COVID-19 hotspot in a retirement home in Hamburg: Prevention concept, case fatality rate and post-mortem findings. Rechtsmedizin.

[72] Schaller T., Hirschbühl K., Burkhardt K., Braun G., Trepel M., Märkl B., Claus R. (2020). Postmortem Examination of Patients With COVID-19. JAMA.

[73] Lindner D., Fitzek A., Bräuninger H., Aleshcheva G., Edler C., Meissner K., Scherschel K., Kirchhof P., Escher F., Schultheiss H.-P. (2020). Association of Cardiac Infection With SARS-CoV-2 in Confirmed COVID-19 Autopsy Cases. JAMA Cardiol..

[74] Wagner W.L., Hellbach K., Fiedler M.O., Salg G.A., Wehrse E., Ziener C.H. (2020). Mikrovaskuläre Veränderungen bei COVID-19. Radiologe.

[75] Bösmüller H., Traxler S., Bitzer M., Häberle H., Raiser W., Nann D., Frauenfeld L., Vogelsberg A., Klingel K., Fend F. (2020). The evolution of pulmonary pathology in fatal COVID-19 disease: An autopsy study with clinical correlation. Virchows Archiv.

[76] Bulfamante G.P., Perrucci G.L., Falleni M., Sommariva E., Tosi D., Martinelli C., Songia P., Poggio P., Carugo S., Pompilio G. (2020). Evidence of SARS-CoV-2 Transcriptional Activity in Cardiomyocytes of COVID-19 Patients without Clinical Signs of Cardiac Involvement. Biomedicines.

[77] Sonzogni A., Previtali G., Seghezzi M., Alessio M.G., Gianatti A., Licini L., Morotti D., Zerbi P., Carsana L., Rossi R. (2020). Liver histopathology in severe COVID 19 respiratory failure is suggestive of vascular alterations. Liver Int..

[78] Carsana L., Sonzogni A., Nasr A., Rossi R.S., Pellegrinelli A., Zerbi P., Rech R., Colombo R., Antinori S., Corbellino M. (2020). Pulmonary post-mortem findings in a series of COVID-19 cases from northern Italy: A two-centre descriptive study. Lancet Infect. Dis..

[79] Cipolloni L., Sessa F., Bertozzi G., Baldari B., Cantatore S., Testi R., D’Errico S., Di Mizio G., Asmundo A., Castorina S. (2020). Preliminary Post-Mortem COVID-19 Evidence of Endothelial Injury and Factor VIII Hyperexpression. Diagnostics.

[80] Tombolini A., Scendoni R. (2020). SARS-CoV-2-related deaths in routine forensic autopsy practice: Histopathological patterns. Int. J. Leg. Med..

[81] Falasca L., Nardacci R., Colombo D., Lalle E., Di Caro A., Nicastri E., Antinori A., Petrosillo N., Marchioni L., Biava G. (2020). Postmortem Findings in Italian Patients With COVID-19: A Descriptive Full Autopsy Study of Cases with and Without Comorbidities. J. Infect. Dis..

[82] Roncati L., Ligabue G., Nasillo V., Lusenti B., Gennari W., Fabbiani L., Malagoli C., Gallo G., Giovanella S., Lupi M. (2020). A proof of evidence supporting abnormal immunothrombosis in severe COVID-19: Naked megakaryocyte nuclei increase in the bone marrow and lungs of critically ill patients. Platelets.

[83] Bussani R., Schneider E., Zentilin L., Collesi C., Ali H., Braga L., Volpe M.C., Colliva A., Zanconati F., Berlot G. (2020). Persistence of viral RNA, pneumocyte syncytia and thrombosis are hallmarks of advanced COVID-19 pathology. EBioMedicine.

[84] Damiani S., Fiorentino M., De Palma A., Foschini M.P., Lazzarotto T., Gabrielli L., Viale P.L., Attard L., Riefolo M., D’Errico A. (2021). Pathological post-mortem findings in lungs infected with SARS-CoV-2. J. Pathol..

[85] Titi L., Magnanimi E., Mancone M., Infusino F., Coppola G., Del Nonno F., Colombo D., Nardacci R., Falasca L., D’Amati G. (2021). Fatal Takotsubo syndrome in critical COVID-19 related pneumonia. Cardiovasc. Pathol..

[86] Del Nonno F., Colombo D., Nardacci R., Falasca L. (2021). Fatal pulmonary arterial thrombosis in a COVID-19 patient, with asymptomatic history, occurred after swab negativization. Thromb. J..

[87] Basso C., Leone O., Rizzo S., De Gaspari M., Van Der Wal A.C., Aubry M.-C., Bois M.C., Lin P.T., Maleszewski J.J., Stone J.R. (2020). Pathological features of COVID-19-associated myocardial injury: A multicentre cardiovascular pathology study. Eur. Hear. J..

[88] Chmielik E., Jazowiecka-Rakus J., Dyduch G., Nasierowska-Guttmejer A., Michalowski L., Sochanik A., Ulatowska-Bialas M. (2021). COVID-19 Autopsies: A Case Series from Poland. Pathobiol..

[89] Cîrstea A.-E., Buzulică R.L., Pirici D., Ceauşu M.C., Iman R.V., Gheorghe O.-M., Neamţu S.D., Stanca L., Ene R., Kumar-Singh S. (2020). Histopathological findings in the advanced natural evolution of the SARS-CoV-2 infection. Rom. J. Morphol. Embryol..

[90] Popa M., Deacu S., Candea L., Comanici S., Pricop S., Mocanu L., Gheju A., Tabirca D. (2020). Virus-associated hemophagocytic lymphohistiocytosis—the severe course expression in sars-cov-2 infection?. Rom. J. Leg. Med..

[91] Oprinca G.-C., Muja L.-A. (2021). Postmortem examination of three SARS-CoV-2-positive autopsies including histopathologic and immunohistochemical analysis. Int. J. Leg. Med..

[92] Kogan E.A., Berezovsky Y.S., Kukleva A.D., Kurilina E.V., Semenova L.A., Blagova O.V., Zharkov N.V. (2020). [Lymphocytic myocarditis in patients with COVID-19 (4 autopsy cases)]. Arkh Patol.

[93] Kovylina M.K., Astakhova O.A., Zayratyants O.Z., Prilepskaya E.P., Reshetov L.R., Kolontarev K.K., Pushkar D.P. (2020). Acute kidney injury in COVID-19: Clinical and morphological comparisons based on autopsy data. Urology.

[94] Conde P.N., Monraval P.A., Medina C.M., Sánchez A.J., Teruel J.C.A., Marco J.F., Santos V.P., Aranda E.M. (2020). Autopsy findings from the first known death from Severe Acute Respiratory Syndrome SARS-CoV-2 in Spain|Autopsia clínica en síndrome respiratorio agudo severo por SARS-CoV-2. Primer fallecido conocido en España. Rev. Española Patol..

[95] Yan L., Mir M., Sanchez P., Beg M., Peters J., Enriquez O., Gilbert A. (2020). COVID-19 in a Hispanic Woman: Autopsy Report with Clinical Pathological Correlation. Arch. Pathol. Lab. Med..

[96] Barca L.V., Cloquell I.T., Cereceda J.H., De Ibarra J.I.S. (2020). An unexplained death after routine cardiac surgery: How long have we dealt with coronavirus disease 2019?. Interact. Cardiovasc. Thorac. Surg..

[97] Hellman U., Karlsson M.G., Engström-Laurent A., Cajander S., Dorofte L., Ahlm C., Laurent C., Blomberg A. (2020). Presence of hyaluronan in lung alveoli in severe Covid-19: An opening for new treatment options?. J. Biol. Chem..

[98] Suess C., Hausmann R. (2020). Gross and histopathological pulmonary findings in a COVID-19 associated death during self-isolation. Int. J. Leg. Med..

[99] Aguiar D., Lobrinus J.A., Schibler M., Fracasso T., Lardi C. (2020). Inside the lungs of COVID-19 disease. Int. J. Leg. Med..

[100] Menter T., Haslbauer J.D., Nienhold R., Savic S., Hopfer H., Deigendesch N., Frank S., Turek D., Willi N., Pargger H. (2020). Post-mortem examination of COVID19 patients reveals diffuse alveolar damage with severe capillary congestion and variegated findings of lungs and other organs suggesting vascular dysfunction. Histopathology.

[101] Nienhold R., Ciani Y., Koelzer V.H., Tzankov A., Haslbauer J.D., Menter T., Schwab N., Henkel M., Frank A., Zsikla V. (2020). Two distinct immunopathological profiles in autopsy lungs of COVID-19. Nat. Commun..

[102] Varga Z., Flammer A.J., Steiger P., Haberecker M., Andermatt R., Zinkernagel A.S., Mehra M.R., Schuepbach R.A., Ruschitzka F., Moch H. (2020). Endothelial cell infection and endotheliitis in COVID-19. Lancet.

[103] Schweitzer W., Ruder T., Baumeister R., Bolliger S., Thali M., Meixner E., Ampanozi G. (2020). Implications for forensic death investigations from first Swiss post-mortem CT in a case of non-hospital treatment with COVID-19. Forensic Imaging.

[104] Seetulsingh P., Kannangara C.I., Richman P. (2020). Undetectable SARS-CoV-2 in a nasopharyngeal swab but persistent viral RNA from deep lung swabs: Findings from an autopsy. BMJ Case Rep..

[105] Dolhnikoff M., Duarte-Neto A.N., Monteiro R.A.D.A., Da Silva L.F.F., De Oliveira E.P., Saldiva P.H.N., Mauad T., Negri E.M. (2020). Pathological evidence of pulmonary thrombotic phenomena in severe COVID-19. J. Thromb. Haemost..

[106] Duarte-Neto A.N., Monteiro R.A.D.A., Da Silva L.F.F., Malheiros D.M.A.C., De Oliveira E.P., Theodoro-Filho J., Pinho J.R.R., Gomes-Gouvêa M.S., Salles A.P.M., De Oliveira I.R.S. (2020). Pulmonary and systemic involvement in COVID-19 patients assessed with ultrasound-guided minimally invasive autopsy. Histopathology.

[107] Santana M.F., Pivoto G., Alexandre M.A.A., Baía-Da-Silva D.C., Borba M.G.D.S., Val F.A., Brito-Sousa J.D., Melo G.C., Monteiro W.M., Souza J.V.B. (2020). Confirmed Invasive Pulmonary Aspergillosis and COVID-19: The value of postmortem findings to support antemortem management. Rev. da Soc. Bras. de Med. Trop..

[108] Miggiolaro A.F.R.D.S., Junior J.D.S.M., de Paula C.B.V., Nagashima S., Malaquias M.A.S., Carstens L.B., Moreno-Amaral A.N., Baena C.P., de Noronha L. (2020). Covid-19 cytokine storm in pulmonary tissue: Anatomopathological and immunohistochemical findings. Respir. Med. Case Rep..

[109] González T.M., de Amat J.D.H.M., Martínez Y.F., Oliva L.L., Cruz L.D.R., Gómez Y.T. (2020). Experience in autopsies of deceased with covid-19 in the central military hospital “dr. Luis díaz soto.” Rev. Cuba Med. Mil..

[110] Barton L.M., Duval E.J., Stroberg E., Ghosh S., Mukhopadhyay S. (2020). COVID-19 Autopsies, Oklahoma, USA. Am. J. Clin. Pathol..

[111] Elsoukkary S.S., Mostyka M., Dillard A., Berman D.R., Ma L.X., Chadburn A., Yantiss R.K., Jessurun J., Seshan S.V., Borczuk A.C. (2021). Autopsy Findings in 32 Patients with COVID-19: A Single-Institution Experience. Pathobiology.

[112] Craver R., Huber S., Sandomirsky M., McKenna D., Schieffelin J., Finger L. (2020). Fatal Eosinophilic Myocarditis in a Healthy 17-Year-Old Male with Severe Acute Respiratory Syndrome Coronavirus 2 (SARS-CoV-2c). Fetal Pediatr. Pathol..

[113] Fox S.E., Akmatbekov A., Harbert J.L., Li G., Brown J.Q., Heide R.S.V. (2020). Pulmonary and cardiac pathology in African American patients with COVID-19: An autopsy series from New Orleans. Lancet Respir. Med..

[114] Prilutskiy A., Kritselis M., Shevtsov A., Yambayev I., Vadlamudi C., Zhao Q., Kataria Y., Sarosiek S.R., Lerner A., Sloan J.M. (2020). SARS-CoV-2 infection–Associated hemophagocytic lymphohistiocytosis an autopsy series with clinical and laboratory correlation. Am. J. Clin. Pathol..

[115] Reichard R.R., Kashani K.B., Boire N.A., Constantopoulos E., Guo Y., Lucchinetti C.F. (2020). Neuropathology of COVID-19: A spectrum of vascular and acute disseminated encephalomyelitis (ADEM)-like pathology. Acta Neuropathol..

[116] Bradley B.T., Maioli H., Johnston R., Chaudhry I., Fink S.L., Xu H., Najafian B., Deutsch G., Lacy J.M., Williams T. (2020). Histopathology and ultrastructural findings of fatal COVID-19 infections in Washington State: A case series. Lancet.

[117] Farkash E.A., Wilson A.M., Jentzen J.M. (2020). Ultrastructural Evidence for Direct Renal Infection with SARS-CoV-2. J. Am. Soc. Nephrol..

[118] Al-Dalahmah O., Thakur K.T., Nordvig A.S., Prust M.L., Roth W., Lignelli A., Uhlemann A.-C., Miller E.H., Kunnath-Velayudhan S., Del Portillo A. (2020). Neuronophagia and microglial nodules in a SARS-CoV-2 patient with cerebellar hemorrhage. Acta Neuropathol. Commun..

[119] Santoriello D., Khairallah P., Bomback A.S., Xu K., Kudose S., Batal I., Barasch J., Radhakrishnan J., D’Agati V., Markowitz G. (2020). Postmortem Kidney Pathology Findings in Patients with COVID-19. J. Am. Soc. Nephrol..

[120] Grimes Z., Bryce C., Sordillo E.M., Gordon R.E., Reidy J., Mondolfi A.E.P., Fowkes M. (2020). Fatal Pulmonary Thromboembolism in SARS-CoV-2-Infection. Cardiovasc. Pathol..

[121] Buja L.M., Wolf D.A., Zhao B., Akkanti B., McDonald M., Lelenwa L., Reilly N., Ottaviani G., Elghetany M.T., Trujillo D.O. (2020). The emerging spectrum of cardiopulmonary pathology of the coronavirus disease 2019 (COVID-19): Report of 3 autopsies from Houston, Texas, and review of autopsy findings from other United States cities. Cardiovasc. Pathol..

[122] Barna N., Chapman J., Hutchins K., Garavan F. (2020). Atypical Endovascular Cells in SARS-CoV-2 Pneumonia. Am. J. Forensic Med. Pathol..

[123] Sauter J.L., Baine M.K., Butnor K.J., Buonocore D.J., Chang J.C., Jungbluth A.A., Szabolcs M.J., Morjaria S., Mount S.L., Rekhtman N. (2020). Insights into pathogenesis of fatal COVID-19 pneumonia from histopathology with immunohistochemical and viral RNA studies. Histopathology.

[124] Occidental M., Flaifel A., Lin L.H., Guzzetta M., Thomas K., Jour G. (2021). Investigating the spectrum of dermatologic manifestations in COVID -19 infection in severely ill patients: A series of four cases. J. Cutan. Pathol..

[125] Iuga A.C., Marboe C.C., Yilmaz M.M., Lefkowitch J.H., Gauran C., Lagana S.M. (2020). Adrenal Vascular Changes in COVID-19 Autopsies. Arch. Pathol. Lab. Med..

[126] Kristine E., Konopka M.D., Allecia Wilson M.D., Jeffrey L., Myers M. (2020). Postmortem Lung Findings in an Asthmatic Patient With Coronavirus Disease 2019. Ann. Oncol..

[127] Lacy J.M., Brooks E.G., Akers J., Armstrong D., Decker L., Gonzalez A., Humphrey W., Mayer R., Miller M., Perez C. (2020). COVID-19: Postmortem Diagnostic and Biosafety Considerations. Am. J. Forensic Med. Pathol..

[128] Paniz-Mondolfi A., Bryce C., Grimes Z., Gordon R.E., Reidy J., Lednicky J., Sordillo E.M., Fowkes M. (2020). Central nervous system involvement by severe acute respiratory syndrome coronavirus-2 (SARS-CoV-2). J. Med Virol..

[129] Nuovo G.J., Magro C., Shaffer T., Awad H., Suster D., Mikhail S., He B., Michaille J.-J., Liechty B., Tili E. (2021). Endothelial cell damage is the central part of COVID-19 and a mouse model induced by injection of the S1 subunit of the spike protein. Ann. Diagn. Pathol..

[130] Rapkiewicz A.V., Mai X., Carsons S.E., Pittaluga S., Kleiner D.E., Berger J.S., Thomas S., Adler N.M., Charytan D.M., Gasmi B. (2020). Megakaryocytes and platelet-fibrin thrombi characterize multi-organ thrombosis at autopsy in COVID-19: A case series. EClinicalMedicine.

[131] Solomon I.H., Normandin E., Bhattacharyya S., Mukerji S.S., Keller K., Ali A.S., Adams G., Hornick J.L., Padera R.F., Sabeti P. (2020). Neuropathological Features of Covid-19. N. Engl. J. Med..

[132] Magro C., Mulvey J.J., Berlin D., Nuovo G., Salvatore S., Harp J., Baxter-Stoltzfus A., Laurence J. (2020). Complement associated microvascular injury and thrombosis in the pathogenesis of severe COVID-19 infection: A report of five cases. Transl. Res..

[133] Borczuk A.C., Salvatore S.P., Seshan S.V., Patel S.S., Bussel J.B., Mostyka M., Elsoukkary S., He B., Del Vecchio C., Fortarezza F. (2020). COVID-19 pulmonary pathology: A multi-institutional autopsy cohort from Italy and New York City. Mod. Pathol..

[134] Attoh S.A., Hobenu F., Edusei L., Agyeman-Bediako K., Laryea C.T., Nyarko E.O., Amedi M.K., Asmah R.H., Asumanu E., McAddy M. (2020). Postmortem diagnosis of COVID-19: Antemortem challenges of three cases at the 37 Military Hospital, Accra, Ghana. Afr. J. Lab. Med..

[135] Khaba M.C., Ngale T.C., Madala N. (2020). COVID-19 in an HIV-infected patient. Lessons learned from an autopsy case. Int. J. Infect. Dis..

[136] Ministry of Health (2020). Emergency Indications Related to the COVID-19 Epidemic Concerning the Funeral Sector, Cemetery, and Cremation. http://www.salute.gov.it/portale/nuovocoronavirus/archivioNormativaNuovoCoronavirus.jsp?lingua=italiano&iPageNo=1&cPageNo=1.

[137] Calabrese F., Pezzuto F., Fortarezza F., Boscolo A., Lunardi F., Giraudo C., Cattelan A., Del Vecchio C., Lorenzoni G., Vedovelli L. (2021). Machine learning-based analysis of alveolar and vascular injury in SARS-CoV -2 acute respiratory failure. J. Pathol..

[138] Bai H.X., Hsieh B., Xiong Z., Halsey K., Choi J.W., Tran T.M.L., Pan I., Shi L.-B., Wang D.-C., Mei J. (2020). Performance of Radiologists in Differentiating COVID-19 from Non-COVID-19 Viral Pneumonia at Chest CT. Radiology.

[139] Dehingia N., Raj A. (2021). Sex differences in COVID-19 case fatality: Do we know enough?. Lancet Glob. Heal..

[140] Lipsitch M., Cohen T., Cooper B., Robins J.M., Ma S., James L., Gopalakrishna G., Chew S.K., Tan C.C., Samore M.H. (2003). Transmission Dynamics and Control of Severe Acute Respiratory Syndrome. Sci..

[141] Chong P.Y., Chui P., Ling A.E., Franks T.J., Tai D.Y.H., Leo Y.S., Kaw G.J.L., Wansaicheong G., Chan K.P., Oon L.L.E. (2004). Analysis of Deaths During the Severe Acute Respiratory Syndrome (SARS) Epidemic in Singapore: Challenges in Determining a SARS Diagnosis. Arch. Pathol. Lab. Med..

[142] Zhao G., Jiang Y., Qiu H., Gao T., Zeng Y., Guo Y., Yu H., Li J., Kou Z., Du L. (2015). Multi-Organ Damage in Human Dipeptidyl Peptidase 4 Transgenic Mice Infected with Middle East Respiratory Syndrome-Coronavirus. PLoS ONE.

[143] Sperhake J.-P. (2020). Autopsies of COVID-19 deceased? Absolutely!. Leg. Med..

[144] Von Stillfried S., Bülow R.D., Röhrig R., Knüchel-Clarke R., Boor P., Tholen P., Nöthel B., Wienströer J., Majeed R., Uhlig S. (2020). Autopsy registry can facilitate COVID -19 research. EMBO Mol. Med..

[145] Gammazza A.M., Légaré S., Bosco G.L., Fucarino A., Angileri F., De Macario E.C., Macario A.J., Cappello F. (2020). Human molecular chaperones share with SARS-CoV-2 antigenic epitopes potentially capable of eliciting autoimmunity against endothelial cells: Possible role of molecular mimicry in COVID-19. Cell Stress Chaperon-.

[146] European Centre for Disease Prevention and Control (2020). Guidance for wearing and removing personal protective equipment in healthcare settings for the care of patients with suspected or confirmed COVID-19. Eur. Cent Dis. Prev. Control. Guidel..

[147] Keresztesi A.-A., Perde F., Ghita-Nanu A., Radu C.-C., Negrea M., Keresztesi G. (2020). Post-Mortem Diagnosis and Autopsy Findings in SARS-CoV-2 Infection: Forensic Case Series. Diagnostics.

[148] Pomara C., Salerno M., Sessa F., Esposito M., Barchitta M., Ledda C., Grassi P., Liberto A., Mattaliano A., Rapisarda V. (2021). Safe Management Strategies in Clinical Forensic Autopsies of Confirmed COVID-19 Cases. Diagnostics.

[149] Carpenito L., D’Ercole M., Porta F., Di Blasi E., Doi P., Fagara G.R., Rey R., Bulfamante G. (2020). The autopsy at the time of SARS-CoV-2: Protocol and lessons. Ann. Diagn. Pathol..

[150] Di Giorgio S., Martignano F., Torcia M.G., Mattiuz G., Conticello S.G. (2020). Evidence for host-dependent RNA editing in the transcriptome of SARS-CoV-2. Sci. Adv..

[151] Bogdanović M., Atanasijević T., Popović V., Mihailović Z., Radnić B., Durmić T. (2021). Is the role of forensic medicine in the covid-19 pandemic underestimated?. Forensic Sci. Med. Pathol..

[152] De Cock K.M., Zielinski-Gutiérrez E., Lucas S.B. (2019). Learning from the Dead. N. Engl. J. Med..

